# Use of irradiated autologous bone in joint sparing endoprosthetic femoral replacement tumor surgery

**DOI:** 10.4103/0019-5413.77137

**Published:** 2011

**Authors:** Sridhar Vijayan, William Bartlett, Robert Lee, Peter Ostler, Gordon W Blunn, Stephen R Cannon, Timothy WR Briggs

**Affiliations:** Royal National Orthopaedic Hospital, Stanmore, HA7 4LP, UK; 1 Mount Vernon Hospital, Northwood, HA6 2RN, UK; 2 University College London and Royal National Orthopaedic Hospital, Stanmore, HA7 4LP, UK

**Keywords:** Bone tumor, femoral tumors, femoral reconstruction, joint preservation, irradiated autologous bone

## Abstract

**Background:**

Joint preservation is usually attempted in cases of bone tumors, though insufficient bone following tumour resection may prevent fixation of conventional joint sparing prosthesis. To preserve the hip joint in skeletally immature patients, we have combined autologous proximal femoral irradiation and intercalary re-implantation with custom made distal femoral replacements.

**Materials and Methods:**

A retrospective cohort study of four patients (aged 4-12 years); in whom irradiated autologous bone was combined with an extendable distal femoral endoprostheses was performed. There were three cases of osteosarcoma and one case of Ewing’s sarcoma.

**Results:**

At a mean follow-up of 70.5 months (range 26-185 months), all four patients were alive without evidence of local recurrence. There was no evidence of metastatic disease in three patients while one patient showed chest metastatic disease at presentation. In all cases, the irradiated segment of bone united with the proximal femur and demonstrated bone ongrowth at the prosthetic collar. There were no cases of loosening or peri-prosthetic fracture. One implant was revised after 14 years following fracture of the extending component of the endoprosthesis.

**Conclusions:**

We report encouraging results utilizing irradiated autologous proximal femoral bone combined with distal femoral replacement in skeletally immature patients.

## INTRODUCTION

Methods of femoral reconstruction following tumor excision include autografts, allografts, rotationplasty, and endoprostheses. Despite promising survival rates, both autograft and allograft reconstructions are associated with a high incidence of fracture, non-union, infection, and loss of articular cartilage.^1^–^5^ Limited donor availability and the potential of disease transfer further reduce the application of allogafts.^3^ Deformity and the need for prosthesis greatly limit the acceptance of rotationoplasty. Endoprosthetic replacements (distal, proximal, and total femoral replacement) are associated with good function in adults and comparable survivorship to amputation.^6^–^9^ However, endoprosthetic replacement is less attractive in children.^10^ Whilst a "growing" prosthesis allows for maintenance of limb length, replacement of the hip joint in the skeletally immature is often associated with later joint subluxation or dislocation.^10^ It is also usually anticipated that children undergoing endoprosthetic replacement that includes the hip will need multiple future revision procedures, this being associated with potential complications of bone loss, soft tissue compromise, and infection.

Extracorporeal irradiation and re-implantation of resected bone (ECIR) was first described in 1988 by Uyttendaele *et al*.^11^ The procedure has now been widely used in orthopaedic tumor reconstructive surgery and has not been associated with an increased risk of recurrent disease.^12^,^13^ ECIR allows for bone stock build-up and avoids risks of loosening, wear and breakage of a massive endoprosthesis. In response to the high reported incidence of fractures and loss of articular cartilage associated with ECIR, Chen *et al*. developed a technique that combined conventional hip arthroplasty prostheses with extracorporeally irradiated autograft.^14^ As replacement of the hip joint is particularly undesirable in the skeletally immature patient, we have developed a new technique of ECIR combined with growing distal femoral endoprosthetic replacement (DFRIB). Here we report our medium-term results of the procedure that allows retention of the patients native hip in situations where there is insufficient bone following tumor resection to allow fixation of a conventional distal femoral endoprosthesis.

## MATERIALS AND METHODS

Between September 1994 and November 2007, two patients each of either sex aged 4 to 12 years were referred to our unit with extensive malignant tumors of the mid to distal femur [Table 1]. Three (case nos.1, 3 and 4) presented with a painful mass. One patient (case no. 2) presented with a pathological fracture of the mid-femoral diaphysis. Prior to referral, this patient had been managed with a mono-axial external fixator using half pins in the proximal and distal femur. All patients underwent neo-adjuvant chemotherapy at their local oncology unit.

**Table 1:** Patient clinical details prior to surgery

Case	Gender	Age (years)	Site	Diagnosis	Surgical stage
1	F	9	Mid-distal femur, marrow signal change proximal femur	Osteosarcoma	2A
2	M	12	Distal femur. Prior external fixation of femoral fracture	Osteosarcoma	2A
3	F	4	Mid-distal femur + skip lesion proximal femur	Ewings sarcoma	3B
4	M	11	Proximal to distal femoral diaphysis	Osteosarcoma	2A

2A = high grade intra-compartmental lesion without metastasis, 3B = extra-compartmental lesion, any grade, with metastasis

Prior to surgical intervention, all patients had initial local and distal staging studies including plain radiographs, whole femur MRI, bone scan, and CT chest. In all patients, tissue diagnosis was obtained by a CT-guided biopsy performed under general anesthesia. Restaging MRI was performed to assess the effects of chemotherapy on the tumor and allow for accurate planning prior to scheduled surgery.

Patients with mid to distal femoral lesions were considered for DFRIB if the planned excision of the tumor with adequate bone and soft tissue margins was expected to prevent secured fixation of a conventional distal femoral replacement. The length of proximal femur required for adequate fixation of a conventional distal femoral replacement depends of the patients’ size and weight though wherever possible, a stem length of 15 cm or more is used. We therefore considered DFRIB in children in whom after tumor excision, anticipated proximal femoral diaphyseal length would be considerably less than 15 cm. In cases where the residual proximal femur was little shorter, conventional distal femoral replacements were employed extracortical plates or curved cemented stems were used and do not form part of the reported cohort.

**Figure 1:**
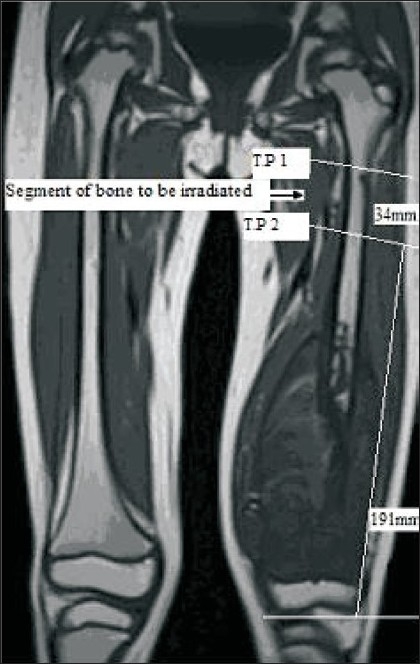
Coronal MRI of both femora (case 3). There is an extensive lesion extending from the mid to distal left femoral diaphysis. An additional lesion in the proximal femoral canal just distal to the lesser trochanter was considered to be a possible skip metastasis. The surgical plan has been marked with dimensions in mm. TP 1 marks the transaction point between bone to be excised below and bone to be harvested and irradiated. TP 2 marks the transaction point of the upper boundary of the bone to be harvested, irradiated and re-implanted

Extracorporeal irradiation and subsequent re-implantation was not performed using diaphyseal bone segments thought to contain the primary tumor, but was used when it was necessary to widen the proximal tumor margin due to margin uncertainty or discontinuous lesions (skip lesions or potential prior surgical contamination). In two cases (cases 1 and 3), the segment of proximal femoral diaphysis harvested for irradiation and subsequent reimplantation was suspected to be disease free, though was affected by extensive proximal femoral bone marrow oedema. In one patient (case 3) with Ewings sarcoma, there was a proximal femoral intramedullary lesion 60 mm from the tip of the greater trochanter with MRI characteristics of an enchondroma, though an atypical skip metastasis was considered as a possibility [Figure 1]. One patient (case 2) had undergone previous external fixation of a pathological spiral fracture of the mid-femur which was thought to have potentially contaminated the proximal femoral diaphysis.

In every case undergoing DFRIB, we aimed to preserve as much disease-free proximal femur as possible, and in all cases retained a minimum of 40 mm of native proximal femur below the tip of greater trochanter. The length of proximal femoral diaphysis that was harvested, irradiated, and re-implanted varied from 34 to 50 mm [Table 2]. Implant stems were designed to be as long as could be accommodated by the combined length of the native proximal femur and irradiated bone composite.

**Table 2:** Characteristics of implants used (n=4)

Case	Growing mechanism	Maximum extension of growing segment (in mm)	Femoral bone length (in mm)	Femoral bone length excised (in mm)	Femoral bone length harvested for irradiation (in mm)	Distance of TP 1 and TP 2 below Tip of greater trochanter (in mm)	% of femur length replaced
1	Minimally invasive grower	100	324	225	45	TP1 44 TP2 89	67
2	Non-invasive grower	90	395	280	40	TP1 100 TP2 140	65
3	Non-invasive grower	50	260	191	34	TP1 54 TP2 88	73
4	Non-invasive grower	90	368	248	50	TP1 92 TP2 142	67

“TP1” indicates the location measured in mm distal from the tip of the greater trochanter, below which the femur is excised. “TP2” indicates the location measured in mm distal from the tip of the greater trochanter, below which a segment of autologous femoral diaphysis is harvested and irradiated before implantation

**Figure 2:**
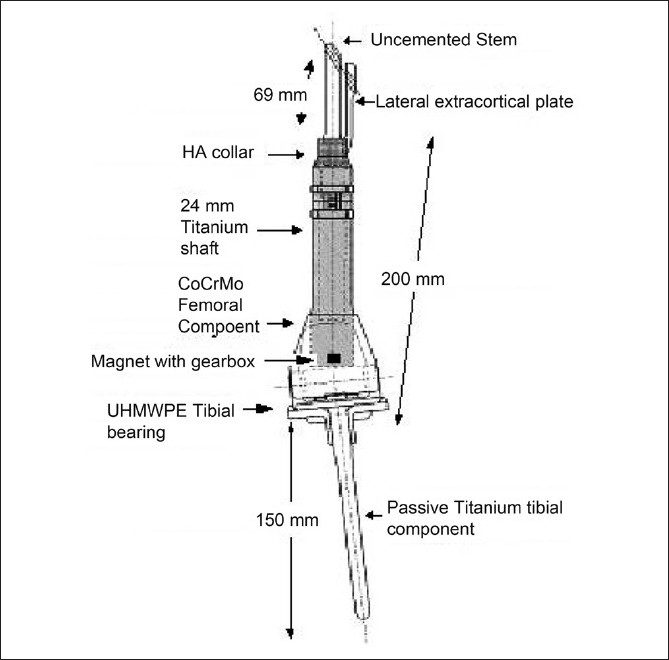
Schematic of distal femoral endoprosthesis used in combination with an irradiated segment of proximal femur as illustrated in Figure 1 (case 3). The prosthesis consists of a HA coated intra-medullary stem with a lateral extracortical plate and locking hole for a 4.5mm cross screw At the base of the stem there is a 18mm HA coated collar. The femoral stem incorporated a magnet and gearbox used allows non-invasive implant extension. The knee joint is a SMILES rotating hinge. The passive growing tibial component is implanted with press-fit only. SMILES = Stanmore Modular Individualized Lower Extremity System; HA = Hydroxyapatite

**Figure 3:**
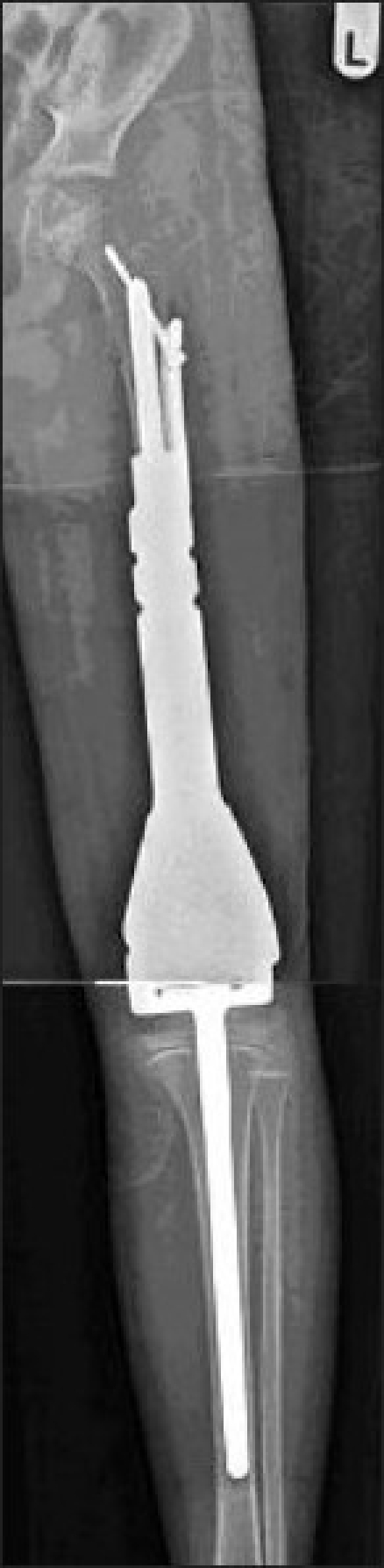
Anteroposterior radiograph of the whole femur and tibia showing the postoperative appearance of the endoprosthesis with irradiated bone composite (case 3)

All implants used were made on a custom basis by Stanmore Implants Worldwide (Middlesex, U.K). The length and diameter of the stem were engineered to the patients remaining bone as determined from preoperative measurement radiographs and MRI. The implants consisted of a proximal uncemented hydroxyapatite (HA) coated femoral stem, a femoral shaft incorporating a growing mechanism, a hinged knee component, and a tibial shaft [Figures 2 and 3]. Early stability was augmented with a single oblique locking screw (4.5-8 mm depending on patient size) passed though a lateral extracortical plate and locked into the intramedullary stem. At the base of the stem is a grooved hydroxyapatite collar of comparable diameter to the patients’ femoral shaft. In one patient (case 1), a “minimally invasive grower” mechanism, designed to be operated by an Allen key, was used. In the three more recently performed cases (cases 2, 3 and 4), "non-invasive grower" mechanisms were incorporated. The knee component was based on the Stanmore modular individualized lower extremity system (SMILES) hinged knee with an ultra-high molecular weight polyethylene bearing (UHMWPE). The knee joint mechanism was a fixed hinge in case 1 and a rotating platform in the later implants. The polished titanium tibial component was designed to be implanted without cement, allowing for potential passive growth at the proximal tibia.^15^

### Operative procedure

All operations were performed by a single surgeon at a tertiary referral institution under general anaesthesia combined with caudal epidural. Intravenous antibiotics were given at the time of induction, and repeated at 8 and 16 hours post-surgery. The patients were positioned supine with a sandbag under the ipsilateral buttock. The incision was made overlying the greater trochanter and extended distally over the lateral thigh, curving to become midline anterior at the knee. The biopsy scar was excised. The femur was then approached by an extended subvastus approach with a lateral parapatellar approach to the knee. The first femoral transection point (TP1) was made at a predetermined level measured from the knee, allowing wide en bloc resection of the tumor and harvesting of the proximal femoral diaphyseal segment to be irradiated. Two small drill holes were made on either side of this planned transaction point in order to facilitate correct orientation of the graft during re-implantation. Imprint specimens were sent from the retained proximal femur. On a side table, a second more distal femoral transection (TP2) was then created to harvest the segment for irradiation from the tumor segment. The positions of TP1 and TP2 are illustrated [Figure 1] and dimensions are shown in Table 2. An imprint biopsy was then also taken from the segment of bone to be irradiated at the level of TP2. The harvested proximal femoral diaphysis was then washed with saline using pulsed lavage, placed in sterile saline, and transported for irradiation. After 90 Grays irradiation, the autologous bone was returned to the operating theatre, typically after a delay of approximately 120 min, during which time the proximal tibia had been prepared to accept the custom-made “passive grower” tibial component. The segment of irradiated bone was then realigned with the native proximal femur and the stem was gently impacted. An implant-specific jig was used to pass the proximal locking screw through the extracortical plate and the lateral cortex of the native proximal femur to the femoral neck. The rest of the implant was then assembled and the wound closed in the standard fashion over a drain.

After surgery, patients were placed in a thigh trough and were subsequently allowed to sit out from their bed at day 3. At this time, physiotherapy was directed at regaining motion at the knee and control of the hip. One patient commenced mobilizing of the hip in the bed using slings and springs. When sufficiently comfortable and stabilized following surgery, patients mobilized non-weight bearing under physiotherapy supervision. Typically, patients were allowed to progress to partial weight bearing at 12 weeks, and fully weight bearing once osteotomy union was evident. They were then followed up regularly and radiographs of the implant were obtained at these intervals whist the patient’s oncologist reviewed surveillance chest X-rays and CT chest.

## RESULTS

The mean follow-up was of 70.5 months (range 26-185 months) with one patient having a follow-up of greater than 41 months. No patients were lost from review. There were no cases of local recurrence. One patient (case 3) had chest metastases on presentation though these responded to chemotherapy and did not progress during the period of her follow-up (49 months). None of the other three patients developed metastatic disease. In all cases, imprint histology from both the retained proximal femur (level TP 1) and the irradiated segment (level TP 2) was negative for tumor.

**Figure 4:**
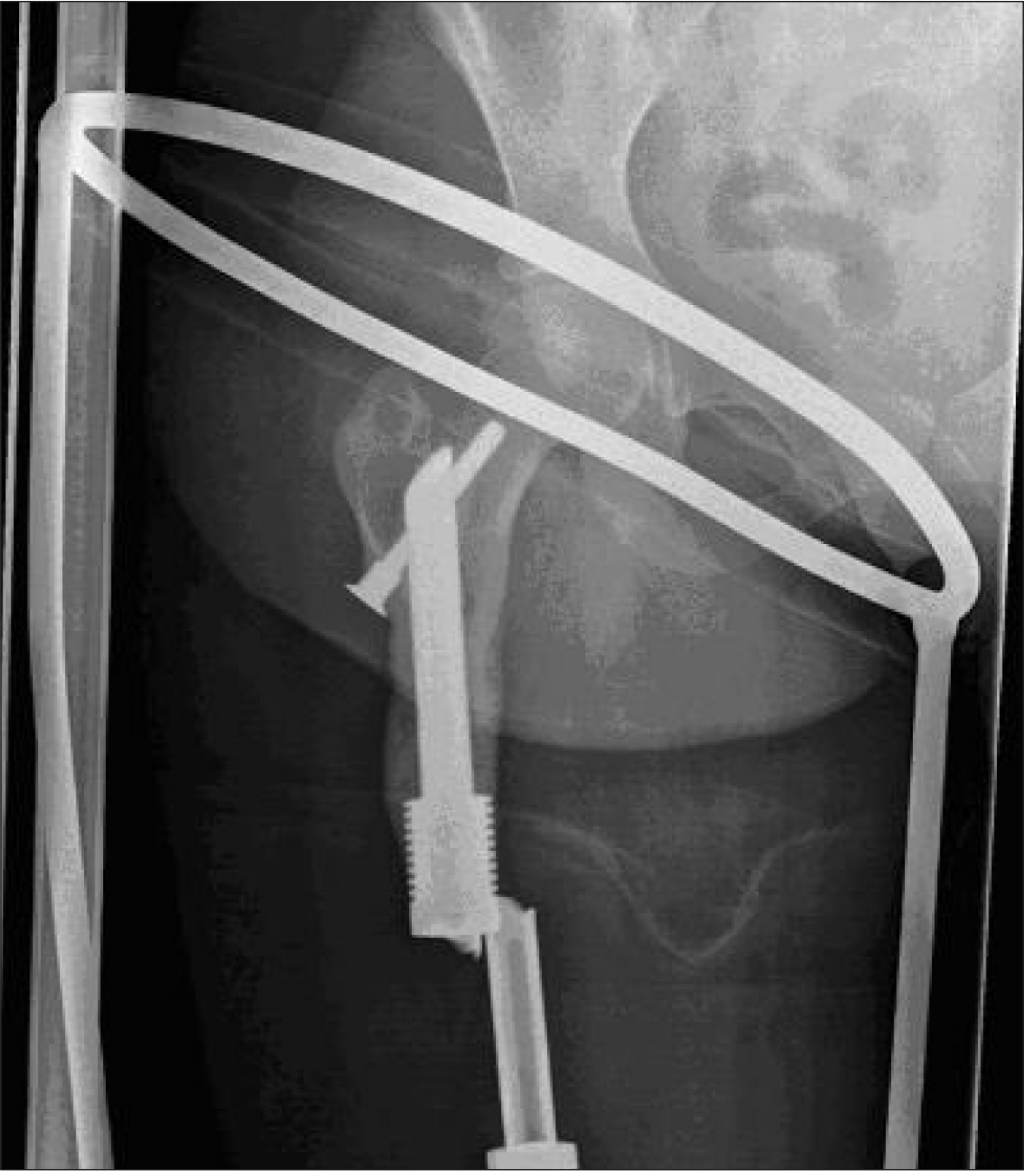
Anteroposterior radiograph of the right proximal femur showing fracture of the extendable femoral shaft component of the growing mechanism 14 years post implantation (case 1). The irradiated segment of autologous bone has fully united proximally and there is significant bone ongrowth around the HA collar

There were no early post-operative complications. All patients were able to mobilize independently by 2 weeks. Partial weight bearing was allowed at 12 weeks. Full weight bearing was allowed after bony union at the osteotomy site. Union was defined when radiographies showed bridging of the osteotomy gap in two planes and was seen after a mean of 29 weeks (range 23-39 weeks). There were no cases of peri-prosthetic fracture, loosening, or deep infection. By 1 year, all patients demonstrated clear radiological evidence of cortical bone on-growth between the irradiated segment of bone and the hydroxyapatite (HA) collar [Figure 4].

Three patients required treatment for flexion deformities at the knee, which developed following lengthening interventions [Table 3]. Case 1 underwent three percutaneous lengthening procedures. The third lengthening was performed at 7 years, when it was necessary to also perform a manipulation under anesthesia with a quadriceps release followed by immobilization in plaster. A further manipulation under anesthesia and reapplication of plaster after 6 weeks allowed the recovery of full knee extension. Two of the other patients (cases 2 and 3) required two re-admissions for in-patient physiotherapy and serial manipulations with casting under general anesthesia. At most recent review, all patients had limb length inequalities of less than 10 mm as measured on blocks.

**Table 3:** Clinical outcomes following surgery

Case	Follow-up (months)	Further procedures	Status	Recurrence	Metastasis disease
1	185	Quadricepsplasty, patella re-alignment, percutaneous leg lengthening, revision of extendable component	Alive	Nil	Nil
2	30	MUA + POP (thrice)	Alive	Nil	Nil
3	41	MUA + POP (twice)	Alive	Nil	No progression
4	26	MUA + POP (once)	Alive	Nil	Nil

MUA = Manipulation under anaesthesia, POP = Plaster of paris

In one patient (case 1), the extending shaft component of the growing mechanism fractured following a fall, 14 years post implantation [Figure 4]. The implant was retained and held with a cemented custom-made sleeve, incorporating the proximal one third of the implant and the remaining femoral shaft.

## DISCUSSION

Limb-salvage surgery incorporating endoprosthetic reconstruction is generally the favored treatment option when managing extensive primary bone tumors of the femur. Good functional outcomes have been reported in adults following distal, proximal, or total femoral endoprosthetic replacements and survival is comparable to amputation.^6^–^9^ However, endoprosthetic replacements that include the hip joint may be troublesome in skeletally immature patients. When performing endoprosthetic reconstruction in children, the implant must be able to accommodate future skeletal growth and allow for a reasonable level of daily activity without significant restrictions. Furthermore, as surgery in combination with neo-adjuvant chemotherapy is potentially curative, implant longevity and bone preservation are critical issues to consider when planning reconstruction.

Specific complications associated with hip replacement in growing children include medial acetabular erosion, osteolysis, joint subluxation/dislocation^16^ progressive head to acetabulum size mis-match, and the unquantified risks associated with lifelong metal ion exposure.^17^ Chandrasekar *et al*.^18^ reported a significant risk of revision surgery secondary due to acetabular erosion in young patients treated with endoprosthetic replacements incorporating monopolar heads. The group advocated the use of either a bipolar or a total hip reconstruction with a metal-on-metal articulation in young patients. In the cases of total proximal femoral replacement, Kampen *et al*.^10^ found that children over the age of 11 had a failure rate of 25% at 10 years and 75% in younger children. In view of the well-established problems associated with hip reconstruction in children undergoing tumor excision, we have developed a technique for treating distal and mid femoral diaphyseal tumors in children that allows preservation of the proximal femoral bone stock and the native hip joint.

Conventional irradiation of bone tumors *in-situ* is limited by the surrounding normal tissue, hence the requirement for a fractionated dose. ECIR allows sparing of the adjacent radiosensitive tissues and delivery of a single dose of irradiation equivalent to two to four times the same dose delivered using fractionated radiation protocols.^19^ In animal models, treatment of between 50 and 70 Gy leads to total destruction of cells within the radiation field^19^,^20^ whilst having minimal impact on matrix proteins such as collagens and bone morphogenic protein.^21^ In the described series there were no cases of local recurrence or new metastatic lesions. However, the purpose of this report was not to describe the efficacy of ECIR, which has been widely demonstrated in both human studies^3^,^11^–^14^,^22^–^25^ and animal models^19^,^20^,^26^ but rather to document the application of the technique in conjunction with extendable distal femoral endoprosthesis in the skeletally immature. One disadvantage of this technique is that the irradiated and reimplanted segment of bone cannot be fully assessed histologically. In all cases, imprint histology from the irradiated segments was clear of tumor cells though these findings do not exclude the presence of skip lesions.

The advantage of using an ECIR over autografting is that the reimplanted segment will be of the appropriate shape and dimensions, and problems such as graft acquisition and storage, immunological complications, and disease transmission are avoided. The adverse effects of high doses of irradiation on the mechanical properties and osteoinductivity of bone graft are well recognized.^27^,^28^ In the animal model, Sugimoto *et al*.^28^ found that an intraoperative dose of 50 Gy to the proximal tibia resulted in a decrease in bone strength that peaked at 24 week, with some return in strength at 52 weeks. Following a literature review of ECIR, Bohm^24^ found an overall reported rate of fractures of 9 of 35 cases. We were therefore reassured to not observe any cases of periprosthetic fracture or implant loosening. Furthermore, all cases demonstrated clear evidence of bone on-growth from the irradiated bone segment to the HA collar and osteotomy union. We aimed to minimize the risk of periprosthetic fracture by the use of locked HA-coated intramedullary fixation that allowed load sharing with the proximal non-irradiated femur. We also consider it to be essential to avoid the re-implantation of bone compromised by osteolytic bone destruction, and to restrict full weight-bearing until bone union at the osteotomy site is observed.

All methods of reconstruction following extensive tumor excision are burdened with a high incidence of complications. We observed knee flexion deformities following lengthening in three patients. This complication is not infrequently observed in children following reconstruction with convention “growing” distal femoral replacement.^29^,^30^ Management is by performing smaller incremental lengthening and adopting a low threshold for manipulation under anesthesia and inpatient physiotherapy. One patient (case 1) sustained an implant fracture at 14 years post-implantation. We suspect that this was related to an overly long extendable segment in the growing component and implant design has subsequently been modified. The fracture of the implant at that point does however illustrate the strength of the fixation of the HA-coated stem within the composite of irradiated autograft and native proximal femur.

Due to the rarity of patients presenting with suitable lesions, we have only used the technique on a small number of patients and initially chose to perform the procedure in children due to the morbidity of hip replacement and the anticipated better osseo-integration. The technique should however be applicable in adults and other anatomical locations. Many issues relating to the described treatment require further clarification such as the length of time needed to obtain stable bone ongrowth at the autograft, the optimal autograft length, and the dose of radiation required. We have been most encouraged by the medium term results of DFRIB in our carefully selected group of patients though larger reports are needed before the technique can be widely adopted.
